# Sex Disparities in Smoked and Smokeless Tobacco Use Among Displaced Populations in Mizoram, India: A Cross-Sectional Study

**DOI:** 10.3390/ijerph22030318

**Published:** 2025-02-20

**Authors:** Yashika Sharma, Ruth Masterson Creber, Julia Lalmuanpuii, Sakie Zawtha, Beichotha Zawtha, Helimay Chairi, Rodani Zawkhai, Stacey Dai, So Hyeon Bang, Natalie Benda

**Affiliations:** 1 School of Nursing, University of Connecticut, Storrs, CT 06269, USA; 2 School of Nursing, Columbia University, New York, NY 10032, USA; rm3284@cumc.columbia.edu (R.M.C.); sd3730@cumc.columbia.edu (S.D.); sb5069@cumc.columbia.edu (S.H.B.); 3 Ferrando Integrated Women Development Center, Aizawl 796025, India; jlmuanpuii@gmail.com; 4 Health and Hope Myanmar, Aizawl 796001, India; sakie@healthandhope.org (S.Z.); beichotha@healthandhope.org (B.Z.); helimay@healthandhope.org (H.C.); rodani@healthandhope.org (R.Z.)

**Keywords:** tobacco use, smokeless tobacco use, global health, India, sex disparities

## Abstract

Displaced populations face an elevated risk for tobacco use, especially smokeless tobacco (SLT), due to its affordability and acceptability in regions like Mizoram State (India) and Chin State (Myanmar). Despite this, limited research exists on tobacco use patterns and contributing factors among displaced populations. This study aimed to examine smoked tobacco and SLT use among displaced communities in Mizoram, India. We collected data across nine villages using mobile health technology. We conducted logistic regression models to examine the cross-sectional associations between sex and tobacco use. Age was explored as a potential moderator. The analytic sample consisted of 2226 participants, with a mean age of 43 (±16.2) years, 63.1% of whom were women. Approximately 70% of the participants reported using tobacco, with SLT being the most common form (46.3%). Women were 57% less likely to use tobacco than men, but women aged 55 and older had twice the odds of using tobacco compared to men in the same age group. Additionally, women had nearly 71 times higher odds of using SLT compared to smoked tobacco than men. The findings underscore the need for culturally sensitive interventions targeting SLT use among women. Future research should explore the drivers of this disparity to guide public health strategies.

## 1. Introduction

Tobacco use is one of the leading causes of preventable deaths worldwide, contributing to a variety of health conditions, including cardiovascular disease, cancer, and respiratory conditions. Approximately 1.25 billion adults worldwide use tobacco, with a global adult smoking prevalence of 32.6% for men and 6.5% for women [1,2]. The decline in smoking prevalence has been uneven across different regions and socioeconomic groups, with high-income countries seeing the largest reductions in tobacco use, and low- and middle-income countries showing slower progress [2]. While global public health efforts have contributed to a reduction in smoking prevalence in many regions, tobacco use continues to pose a significant public health burden, particularly in low- and middle-income countries.

Smoked tobacco is the most commonly recognized form of tobacco use. However, smokeless tobacco also has significant health implications, particularly in regions where these products are culturally ingrained. India, in particular, has one of the highest rates of smokeless tobacco use, with 185.8 million users, representing 68% of all users worldwide [3]. Some of the most widely used products include *khaini* (sun-dried tobacco mixed with lime), *gutkha* (a mix of crushed areca nut, tobacco, and other ingredients), and *betel nut* (also known as “paan with tobacco”) [4]. The products are deeply embedded in social and cultural traditions, making it challenging to reduce their use despite growing awareness of the health risks. The northeastern region of India stands out as a high-burden area for both smoked and smokeless tobacco use. It also has one of the highest prevalences of tobacco consumption, particularly among men and those employed in labor-intensive occupations [5,6].

The growing prevalence of smokeless tobacco use in Northeast India presents a unique challenge for public health. Studies have consistently linked smokeless tobacco to increased risks of cardiovascular disease, oral cancer, and other adverse health outcomes [7]. A case-control study in Bangladesh found that among people who had ever used smokeless tobacco, there was a four-fold increased risk of coronary heart disease (adjusted OR = 4.0, 95% CI: 2.0–8.1) compared to non-users, even after adjusting for smoking and other confounding factors [8]. Smokeless tobacco products contain harmful chemicals like nicotine, tobacco-specific nitrosamines, and heavy metals. These substances contribute to atherosclerosis, hypertension, and an elevated risk of stroke, placing tobacco users at a higher risk for cardiovascular events [9]. In addition to cardiovascular issues, smokeless tobacco use significantly raises the risk of oral cancers, and is positively associated with cancers in the pancreas, pharynx, and esophagus [10,11]. Contrary to common belief, smokeless tobacco use during pregnancy is also associated with adverse birth outcomes, including low birth weight and preterm birth, restricted head growth, placental problems, increased risk of stillbirth, and increased risk of miscarriage [12,13]. Given the widespread use of smokeless tobacco products, and corresponding severity of health outcomes, addressing smokeless tobacco use in Northeast India is critical for reducing the overall burden of disease and improving public health outcomes.

Smoking rates are higher among refugee populations compared to non-refugees [14,15,16]. Refugees are at risk for higher rates of chronic diseases [17], mental health issues [18], and poorer self-rated health compared to non-refugee populations [19]. Many refugees may also resort to tobacco use as a way to cope with stress and trauma [20]. This increased tobacco use can exacerbate existing health issues and contribute to poor long-term health outcomes. Furthermore, the health impacts of tobacco use may be compounded by limited access to healthcare services and preventive care among refugee populations [21].

Given the significant health disparities and unique challenges faced by refugee populations, understanding and addressing tobacco use in this vulnerable group is important. However, despite the evident need, there remains a considerable research gap in this area. While studies have documented higher smoking rates among some refugee populations, comprehensive data on tobacco use patterns and the factors that predict tobacco use among refugees are still limited.

Our study focuses on assessing the prevalence of smoked and smokeless tobacco use among displaced communities Northeast India, specifically in the Mizoram State. We deliberately use the term “displaced persons” to describe this population. Although they meet the criteria for “refugees” under the 1951 Refugee Convention, India, as a non-signatory, does not formally classify them as “refugees” [22,23]. Furthermore, the designation of “displaced persons” is particularly significant as it often determines the level of protection they can expect [24]. The lack of identification documents and medical records restricts their access to humanitarian aid and quality healthcare [24]. The term “displaced person” also aligns with United Nations Educational, Scientific and Cultural Organization (UNESCO)’s broader definition, which includes both internal and cross-border displacement [25].

Nearly 50,000 Burmese persons have been displaced to the area following the 2021 military coup d’etat in Myanmar. This group consists mostly of persons displaced from Western Myanmar’s ethnic minority Chin State. Previous studies have described challenges to quantifying tobacco use in Myanmar as a whole, with a paucity of data on tobacco use in the Chin State [26,27,28,29] The Chin people are an oppressed, ethnic minority group, and are now facing the extreme stressors of displacement, underscoring the importance of quantifying health issues and developing culturally appropriate public health interventions. Understanding the prevalence and patterns of tobacco use will inform appropriate strategies for cessation and health promotion among displaced populations.

## 2. Methods

The mHealth for Mothers in Mizoram (mUMM) study aims to improve maternal healthcare in Mizoram, India, through the use of mobile health (mHealth) technology. The goal of this ongoing study is to develop and implement innovative mobile health solutions for displaced populations. This initiative involves a collaborative effort between Columbia University School of Nursing and two India-based organizations: the Ferrando Integrated Women’s Development Centre (FIWDC) and Health and Hope (HH). FIWDC is an Indian non-profit with nearly 20 years of experience providing health and social services to vulnerable women and children of the Mizo tribe. Most of the staff at FIWDC and HH are members of the Mizo (local to India) and Chin (local to Myanmar) tribes, respectively, providing essential linguistic and cultural expertise to adapt and implement interventions that are socially and culturally relevant.

### 2.1. Study Design and Sampling

The study employed a cross-sectional design and convenient sample approach to target displaced populations attending mobile health clinics in Mizoram, India. All adults aged 18 and older in the displaced populations were eligible for inclusion.

The mHealth data collection infrastructure established through the mMUMM study allowed us to expand population health surveillance to the general population and collect data examining tobacco use among displaced people living in Mizoram. Since our analysis focused on adults aged 18 and older, we excluded participants under the age of 18 (*n* = 1556). We further excluded participants with missing data on tobacco product use (*n* = 145), leaving a total of 2226 participants for analysis.

### 2.2. Data Collection

Data collection took place across nine villages in Mizoram, conducted by trained data collectors using mobile phones. Data were collected during periodic mobile health clinics that the organizations conduct as a service to the community in support of their respective missions. The data were collected using KoboToolbox, an open-source tool designed for challenging environments and global contexts. KoboToolbox supports data collection in multiple languages, which facilitated the collection of data in both Mara and Burmese, which was later translated into English.

## 3. Tobacco Use

**Figure 1:**
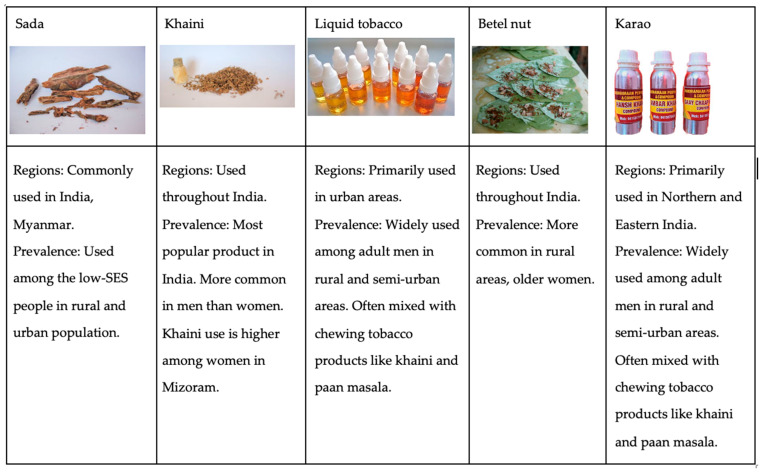
Types of smokeless tobacco (SLT) products commonly used in Mizoram, India.

The sample of participants was drawn from mobile health clinics that were focused on service delivery rather than data collection. To accommodate a low-literacy population and capture the diverse tobacco products used in Northeast India (see Figure 1), we developed brief, straightforward questions in collaboration with local health professionals familiar with the target population, as no existing tools met these needs.

We assessed tobacco use by asking participants, “Do you use tobacco products?” (1 = “Yes”; 0 = “No”). Those who responded “Yes” were asked to specify the type of tobacco products used from the following options: (1) Smoking, (2) Sada, (3) Khaini, (4) Liquid Tobacco, (5) Betel Nut, and (6) Other. Participants who selected “Other” specified the use of “Karao”. This was a select-multiple question, allowing participants to choose more than one option (Figure 1).

We created a composite variable for smokeless tobacco (SLT) that included: sada, khaini, liquid tobacco, betel nut, and karao. We then categorized tobacco use into four groups: (1) Both (i.e., those who used both smoked and SLT products); (2) Smoked (i.e., those who used only smoking tobacco products and no SLT); (3) Smokeless (i.e., those who used at least one SLT product but did not smoke); and (4) No Tobacco Use (i.e., those who reported not using any tobacco products).

## 4. Sociodemographic Characteristics

We primarily assessed age (18–24, 25–34, 35–44, 45–54, and 55+) and sex (male vs. female). There were various other sociodemographic variables collected, such as district and village, which are not the focus of this report.

## 5. Statistical Analysis

All the analyses were performed using R (version 4.3.2). First, we examined the data for patterns, missing values, and outliers. We excluded participants with missing data on tobacco product use (*n* = 145). Next, we calculated means, standard deviations, and proportions to describe the distribution and frequencies of each variable. We then explored the distribution of responses across age and gender categories for different types of tobacco use to identify patterns and inform subsequent analyses. The distribution of responses provided insights before proceeding to hypothesis testing.

We ran logistic regression models to examine the cross-sectional associations between sex (independent variable) and tobacco use (dependent variable), controlling for age. We also performed moderation analysis to examine whether age (18–24, 25–34, 35–44, 45–54, and 55+) moderated the relationship between sex and tobacco use (all forms) as well as sex and SLT use alone. Interaction terms (sex x age categories) were included in the logistic regression models to test for moderation. Statistical significance was set at a *p*-value < 0.05 for all analyses.

To further explore sex disparities in tobacco use, we conducted additional logistic regression models assessing the association between sex and specific tobacco products, including sada, khaini, liquid tobacco, and betel nut, compared to no tobacco use (Supplementary Table S1). Additionally, we performed moderation analysis to assess whether age moderated these associations by incorporating interaction terms between sex and age categories (Supplementary Table S2).

## 6. Results

### 6.1. Demographic Characteristics

Table 1 presents differences in tobacco use and age by sex. The analytic sample included 2226 participants; the mean age of our sample was approximately 43 years (±16.2). A total of 1405 (63.1%) participants were women, and 821 (37%) participants were men. Most of the sample reported using some form of tobacco product (~70.0%). Among those who reported tobacco use, most used SLT (46.3%), followed by both smoked and smokeless (12.4%) and then smoked only (11.1%). In terms of specific SLT products, betel nut was the most common among those using SLT (43.4%) followed by sada (34.8%), khaini (20.4%), and liquid tobacco (20.4%). Notably, most participants reported using more than one type of SLT product. Compared to men, women were younger (44.4 vs. 42.8 years), less likely to use smoked tobacco (28.0% vs. 1.1%) but more likely to use SLT products (21.2% vs. 61.0%).

**Figure 2:**
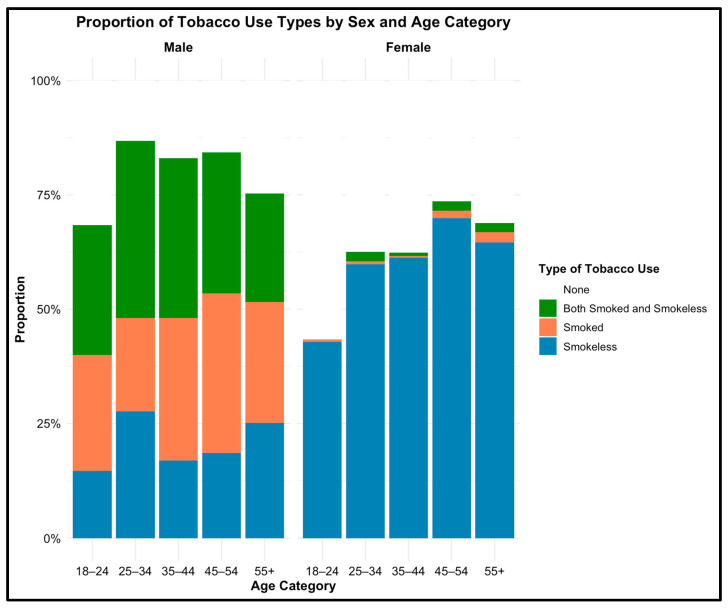
Promotion of tobacco use types by sex and age categories.

Figure 2 illustrates the distribution of responses in the proportion of different types of tobacco use by gender and age. These proportions reflect patterns observed in the data and are not based on statistical testing. These descriptive proportions led us to statistical analyses investigating age as a moderator of the association between sex and tobacco.

Men used more smoked and smokeless tobacco products, especially in the 25–54 age category. In contrast, women predominantly used SLT across all age categories with minimal use of smoked or both types of tobacco products. SLT use among women increases with age, with the highest usage observed in the older age groups (45–54 years: 69.9%; 55+ years: 64.6%). However, among older men, the use of smoked and both smoked and SLT remain more prevalent (Figure 2).

### 6.2. People Who Use Tobacco vs. People Who Do Not

Table 2 presents the results of the logistic regression models examining the association between sex and tobacco use (reference: no tobacco use). After adjusting for age, females were 57% less likely to use tobacco compared to males (AOR = 0.43, 95% CI = 0.35–0.53).

Table 3 shows the results of moderation analyses examining whether age moderated the association between sex and tobacco use. The interaction terms were not statistically significant for the 25–34, 35–44, and 45–54 age groups, indicating no significant differences in tobacco use between males and females in these groups. In the 55+ age group, the interaction term was statistically significant, with females having higher odds of tobacco use compared to males (AOR = 2.02, *p* = 0.03).

### 6.3. SLT vs. Smoked Tobacco

Table 4 shows the results of a logistic regression model examining the association between sex and SLT use. After adjusting for age, females had 70.6 times higher odds of using SLT compared to smoked than males (AOR = 70.6, 95% CI = 42.78–124.92).

Table 5 presents the results of moderation analyses examining whether age moderated the association between sex and SLT use. None of the interaction terms were statistically significant, indicating no significant differences in the relationship between age and SLT use across sex categories.

### 6.4. Tobacco Use by Product Type

Supplementary Table S1 shows the results of logistic regression models examining the association between sex and each specific type of tobacco use. After adjusting for age, women had significantly higher odds of using sada (AOR = 3.22, 95% CI = 2.26–4.59), khaini (AOR = 2.59, 95% CI = 1.31–5.11), and liquid tobacco (AOR = 1.96, 95% CI = 1.26–3.03) compared to males. Conversely, men were significantly more likely to use smoked tobacco (AOR = 0.02, 95% CI = 0.02–0.04). Betel nut use was more evenly distributed between men and women, with a less pronounced sex difference.

Supplementary Table S2 presents the results of logistic regression models examining whether age moderated the relationship between sex and specific types of tobacco use. We found that, generally, older women had higher odds of using SLT compared to younger women and men in the same age group.

## 7. Discussion

The study reports the prevalence of tobacco use, particularly highlighting the sex disparities in SLT use, among the displaced population living in Mizoram, India. Women were 57% less likely to use tobacco compared to men. However, the significant interaction term for the 55+ age group suggests that age moderates the relationship between sex and tobacco use, with women aged 55 and older having twice the odds of using tobacco compared to men in the same age group. Notably, women had nearly 71 times higher odds of using SLT than smoked tobacco, compared to men.

We found that women had lower odds of tobacco use compared to men, but this trend shifted in the older age group, where women aged 55 and older faced double the risk of tobacco use compared to men. Similar trends are observed in Myanmar (the home country of the displaced population in this study), where women are generally less likely to use tobacco compared to men, but smoking is more prevalent among older women [30]. There are several potential explanations for this observation. First, an analysis from the nationally representative sample of the Global Adult Tobacco Survey (GATS; 2009–2010) in India found that younger adults were significantly more willing to quit tobacco use than older adults [31]. Another analysis of the GATS data (2009–2010 and 2016–2020) found that women in the Northeast region had the lowest odds of quitting tobacco compared to women in other parts of India [31]. Similarly, a recent analysis of nationally representative survey Longitudinal Aging Study in India comprising over 38,000 middle-aged and older women found an increased risk of tobacco use among women in the northeastern region, particularly those living in lower socioeconomic areas, rural regions, living alone, or experiencing depressive symptoms [32]. Furthermore, there are generational differences as older women were exposed to different social norms and attitudes towards smoked tobacco during their youth. All these factors could explain why older women in Mizoram are at higher risk of tobacco use compared to older men in Mizoram.

Consistent with the literature, our findings highlight that women are at an alarmingly higher risk of SLT use compared to men. Analysis of nationally representative data from over 93,000 women in the northeastern states of India revealed that SLT use is most prevalent in Mizoram [33]. There are several factors that contribute to this disparity. Tobacco use is deeply ingrained in cultural and traditional practices in Myanmar as well as Mizoram, India (the current location where they are staying as displaced individuals) [28,29,30]. Sociologically, the use of smoked tobacco among women is considered socially inappropriate, as it is not viewed as a feminine activity. However, social norms in India condone women’s use of SLT [34,35]. Additionally, there are long-standing cultural uses of SLT in the region. For instance, khaini is used as an insect repellent to keep flies away from the face and bugs off crops. Both in Myanmar and India, liquid tobacco and betel quid are believed to be a pain reliever for toothaches and serve the purpose of a breath freshener [26,27,28,29]. Similarly, in Myanmar, the use of SLT is particularly common in rural areas where chewing betel liquid is a part of social interaction [28,29]. Both among the Mizo (local to India) and Chin (local to Myanmar) tribes, offering betel leaves or betel nuts is a common part of the traditional hospitality, and is considered disrespectful to refuse the offer.

Lack of access to education and misinformation may be key contributing factors to high SLT use among women, as suggested by previous studies. Furthermore, studies indicate that many women perceive multiple benefits of using SLT. Women from lower socioeconomic backgrounds claim that SLT suppresses their hunger and provides them with energy to engage in heavy labor with limited access to food. Notably, pregnant women also report using SLT, believing that SLT use strengthens teeth and gums during pregnancy [35]. There is also misinformation that SLT use during pregnancy can provide health benefits and relieve common pregnancy-related symptoms, such as nausea, vomiting, and constipation [26,27]. While our study was not able to analyze these factors directly, these contextual insights from the existing literature provide an important lens through which to interpret the findings.

Another important factor to highlight that potentially contributes to the higher SLT use among women in Mizoram is poverty. Using nationally representative data from GATS II (2016–2017), investigators examined tobacco use and socioeconomic variations in tobacco consumption among women and found that SLT was highest among the poorest quintiles. The lower taxation on SLT products, inconsistent with smoked tobacco, is a contributing factor. Indeed, a cross-sectional study conducted among countries that are Parties to the World Health Organization (WHO) Framework Convention on Tobacco Control (FCTC) identified significant disparities in tobacco taxation, with the largest disparity between cigarette and SLT taxation occurring in Southeast Asia, including India [36]. Current SLT control policies in India are viewed as inadequate or poorly implemented with little to no regulatory mechanisms in place, such as licensing requirements and trading standards [37]. This is concerning, as evidence suggests that, like cigarettes, taxes and prices are key factors in controlling the demand for SLT products.

We should also consider the unique stressors related to displacement, including trauma from violence, loss of social support, economic hardship, and poor mental health, all of which can drive tobacco use as a coping mechanism [14,15,16,19,20]. The availability, affordability, and cultural acceptance of SLT in regions like Mizoram may further perpetuate its use. Displaced women are particularly vulnerable in Mizoram as cultural norms stigmatize the use of smoked tobacco while promoting perceived benefits of SLT use among women. Although specific studies examining the prevalence of SLT use among displaced populations are limited, the available research shows similar trends. For instance, a study examining tobacco usage among Bhutanese refugees in the United States found a significantly higher prevalence of SLT use among the refugees than non-Hispanic Caucasians [38]. Notably, the investigators found gender differences among the Bhutanese refugees, where more men smoked cigarettes while more women used SLT, but this trend was not observed among non-Hispanic Caucasians [38].

Even though research on SLT use among refugee communities is scarce, a few studies have attempted to explore the drivers behind this behavior. For instance, a recent study investigating SLT use among refugee communities (including refugees from Myanmar) in San Antonio found that 70% of participants described SLT as a “feel-good” drug [39]. Despite over 80% acknowledging SLT’s harmful effects, including its link to oral cancer, many were unable to quit due to their stressful living conditions. In fact, 64% expressed a desire to quit SLT use, and approximately 36% had attempted to quit but been unsuccessful due to ongoing challenges. These findings underscore the role of displacement-related stress in driving tobacco use, particularly among women, and highlight the need for targeted interventions.

### 7.1. Public Health Implications

Our findings highlight several important implications for future research. There is a critical need to further examine the social determinants of health that impact SLT use among women in displaced communities, particularly in Southeast Asia, where approximately 83% of global SLT users live. Longitudinal studies are essential to understand the long-term impacts of SLT use. A deeper understanding of the contributing factors to this disparity will help identify potential targets for future behavioral or psychosocial interventions aimed at reducing SLT use among women in displaced communities. The cultural uses of SLT, particularly among women, must also be carefully considered in order to design culturally congruent interventions, specifically those that offer safer alternatives to the current perceived benefits of SLT.

The findings from this study also have significant clinical implications. Clinicians working with displaced populations should actively screen for SLT use, particularly among women, and provide education on the associated detrimental health risks. Interventions should consider dual-purpose messaging relevant to SLT use in addition to smoked tobacco. Considering that SLT use is deeply embedded in social settings due to cultural values, community-level interventions should be prioritized for SLT cessation. Community programs that encourage women to participate in group-based SLT cessation efforts may help reduce SLT use among this vulnerable group.

### 7.2. Limitations

There are several limitations that should be taken into consideration when interpreting the findings of the study. First, we utilized a convenience sampling approach. Although this approach was necessary for the inclusion of a hard-to-reach displaced population, it does limit the generalizability of our findings. Second, residual confounding may be present, as we did not assess other social determinants of health (e.g., education, income, social support, depressive symptoms) that have been shown to influence tobacco use. However, this omission is primarily due to the unique context of the displaced population studied. The study population has uniformly low levels of education as education opportunities are extremely limited, with few to no schools beyond the primary level. Additionally, due to their status as displaced persons, they are not legally authorized to work in the current context, and most originate from areas where farming and trading are the most common forms of employment. As such, questions regarding income and employment would not be applicable or meaningful. Given the nature of this displaced population, income and social support are likely to be limited, which may be key contributing factors to the SLT use among women. Third, this study is cross-sectional, so we are unable to infer causality from the observed associations. Fourth, all data are self-reported, which is subject to recall bias. Given the social stigma around smoking among women in India, it is possible that women underreported their tobacco use, particularly smoked tobacco use. Fifth, the study sample is drawn from displaced populations in Mizoram, which limits the generalizability of our findings. However, given that Mizoram has one of the highest prevalences of SLT use among women globally [33,40], our study addresses a critical yet understudied area.

## 8. Conclusions

This study provides valuable insights into the prevalence of tobacco use, particularly SLT use, among displaced populations in Mizoram, India, which is an understudied area. Findings reveal significant sex- and age-related disparities, with older women showing a higher risk of tobacco use than older men. The higher odds of SLT use among women compared to men could underscore the differential influence of cultural, social, and economic factors on SLT use between women and men. Future research is needed to understand the sociocultural factors that contribute to these disparities observed among women. Clinicians should prioritize screening and education on the health risks of SLT use among women in displaced populations. Findings highlight the need for future research to examine the drivers of this disparity and to develop targeted public health interventions for women.

## Figures and Tables

**Table 1:** Differences in Demographic Characteristics Between Men and Women (N = 2226).

Characteristic	Men (*n* = 821)	Women (*n* = 1405)	*p*-Value	Total (N = 2226)
	No. of Participants (%)			No. of Participants (%)
Age (mean (SD))	44.4 (16.2)	42.8 (16.2)	0.025	43.4 (16.2)
Age (years)			0.010	
18–24	95 (11.6)	175 (12.5)		270 (12.1)
25–34	152 (18.5)	344 (24.5)		496 (22.3)
35–44	183 (22.3)	287 (20.4)		470 (21.1)
45–54	172 (21.0)	246 (17.5)		418 (18.8)
55+	219 (26.7)	353 (25.1)		572 (25.7)
Tobacco use	661 (80.5)	895 (63.7)	<0.001	1556 (69.9)
Type of tobacco use			<0.001	
Smoking	230 (28.0)	16 (1.1)		246 (11.1)
Smokeless	174 (21.2)	857 (61.0)		1031 (46.3)
Both smoking and smokeless	255 (31.1)	21 (1.5)		276 (12.4)
None	162 (19.7)	511 (36.4)		673 (30.2)
Type of smokeless tobacco			<0.001	
Sada	81 (12.3)	461 (51.5)		542 (34.8)
Khaini	49 (7.4)	268 (29.9)		317 (20.4)
Liquid Tobacco	51 (7.7)	234 (26.1)		285 (18.3)
Betel Nut	331 (50.1)	345 (38.5)		676 (43.3)
Karao	0 (0.0)	3 (0.3)		3 (0.2)

**Table 2:** Any tobacco product use vs. No tobacco product use: Results of logistic regression models examining the associations between sex and tobacco use (N = 2226).

	Models	Odds Ratio (95% CI)	B	S.E.	Wald	Pseudo-R^2^
Gender (Reference: Male)	Model 1 (OR)	0.42 *** (0.35–0.52)	−0.86	0.10	67.62	0.03
	Model 2 (AOR)	0.43 *** (0.35–0.53)	−0.84	0.10	65.27	0.03

Note. Odds Ratio = OR; Adjusted Odds Ratio = AOR; Confidence Interval = CI; Beta = B; S.E. = Standard Error; Wald = Wald Statistic; Pseudo-R^2^ = Goodness-of-fit statistic. Model 1 is unadjusted. Model 2 is adjusted for age. *** *p* < 0.001

**Table 3:** Any tobacco product use vs. no tobacco product use.

	AOR	95% CI	B	S.E.	Wald	Pseudo-R^2^
Gender (Reference: Male)						
Female	0.35 ***	(0.21–0.59)	−1.04	0.27	14.96	0.05
Age categories (Reference: 18–24)						
25–34	3.23 ***	(1.42–7.25)	1.17	0.33	12.63	
35–44	2.26 **	(1.26–4.05)	0.82	0.3	7.62	
45–54	2.48 **	(1.37–4.52)	0.91	0.3	8.89	
55+	1.45	(0.84–2.45)	0.37	0.27	1.84	
Gender * Age categories						
Female * 25–34	0.67	(0.32–1.41)	−0.4	0.38	1.09	
Female * 35–44	0.95	(0.48–1.91)	−0.05	0.35	0.02	
Female * 45–54	0.7	(0.71–3.02)	0.38	0.37	1.06	
Female * 55+	2.02 *	(1.06–3.88)	0.7	0.33	4.47	

Note. Adjusted Odds Ratio = AOR; Confidence Interval = CI; Beta = B; S.E. = Standard Error; Wald = Wald Statistic; Pseudo-R^2^ = Goodness-of-fit statistic. * *p* < 0.05, ** *p* < 0.01, *** *p* < 0.001.

**Table 4:** Smokeless tobacco use vs. smoked: Results of logistic regression models examining the associations between gender and smokeless tobacco use (N = 1277).

	Models	Odds Ratio (95% CI)	B	S.E.	Wald	Pseudo-R^2^
Gender (Reference: Male)	Model 1 (OR)	70.80 *** (42.89–125.19)	4.26	0.27	246.06	0.431
	Model 2 (AOR)	70.64 *** (42.78–124.92)	4.26	0.27	245.63	0.432

Note. Odds Ratio = OR; Adjusted Odds Ratio = AOR; Confidence Interval = CI; Beta = B; S.E. = Standard Error; Wald = Wald Statistic; Pseudo-R^2^ = Goodness-of-fit statistic. Model 1 is unadjusted. Model 2 is adjusted for age. *** *p* < 0.001.

**Table 5:** Smokeless tobacco use vs. smoked: Results of logistic regression models examining age as a moderator of the associations between sex and tobacco use (N = 1277).

	AOR	95% CI	B	S.E.	Wald	Pseudo-R^2^
Gender (Reference: Male)						
Female	128.57 ***	(24.19–2393.32)	4.86	1.06	20.94	0.45
Age categories (Reference: 18–24)						
25–34	2.32 *	(1.05–5.30)	0.84	0.41	4.20	
35–44	0.93	(0.43–2.09)	−0.07	0.40	0.03	
45–54	0.91	(0.42–2.04)	−0.09	0.40	0.05	
55+	1.63	(0.77–3.53)	0.49	0.39	1.59	
Gender * Age categories						
Female * 25–34	0.59	(0.03–7.09)	−0.53	1.3	0.16	
Female * 35–44	2.52	(0.09–69.15)	0.92	1.48	0.39	
Female * 45–54	0.63	(0.03–5.09)	−0.47	1.2	0.15	
Female * 55+	0.23	(0.01–1.56)	−1.45	1.14	1.64	

Note. Adjusted Odds Ratio = AOR; Confidence Interval = CI; Beta = B; S.E. = Standard Error; Wald = Wald Statistic; Pseudo-R^2^ = Goodness-of-fit statistic. * *p* < 0.05, *** *p* < 0.001.

## Data Availability

Data is unavailable due to privacy restrictions.

## Supplementary Materials

The following supporting information can be downloaded at: https://www.mdpi.com/article/10.3390/ijerph22030318/s1, Table S1: Smoking and Different Types of Smokeless Tobacco vs. No Tobacco Use: Results of Logistic Regression Models Examining the Associations Between Gender and Tobacco Use (N = 2226); Table S2: Smoking and Different Types of Smokeless Tobacco vs. No Tobacco Use: Results of Logistic Regression Models Examining Age as a Moderator of the Associations Between Gender and Tobacco Use (N = 2226).
