# Supplementary material for: Sex Disparities in Smoked and Smokeless Tobacco Use Among Displaced Populations in Mizoram, India: A Cross-Sectional Study
Source: Int J Environ Res Public Health. 2025 Feb 20;22(3):318. doi: 10.3390/ijerph22030318 (PMC11942137; doi:10.3390/ijerph22030318)
Supplement: Supplementary file 1 [file ijerph-22-00318-s001.zip › ijerph-3318583-supplementary.pdf]

**Table S1.** Smoking and Different Types of Smokeless Tobacco vs. No Tobacco Use: Results of Logistic Regression Models Examining the Associations Between Gender and Tobacco Use (N=2226).

| | **Model 1** | **Model 2** |
|---|---|---|
| **Gender (Reference: Male)** | **OR (95% CI)** | **AOR (95% CI)** |
| Smoking | 0.02 (0.02–0.04) | 0.02 (0.02–0.04) |
| Sada | 3.21 (2.26–4.58) | 3.22 (2.26–4.59) |
| Khainie | 2.62 (1.33–5.16) | 2.59 (1.31–5.11) |
| Liquid Tobacco | 1.63 (1.08–2.47) | 1.96 (1.26–3.03) |
| Betel Nut | 0.52 (0.37–0.71) | 0.50 (0.36–0.69) |

Note. Odds Ratio = OR; Adjusted Odds Ratio = AOR; Confidence Interval = CI. Model 1 is unadjusted. Model 2 is adjusted for age.

**Table S2.** Smoking and Different Types of Smokeless Tobacco vs. No Tobacco Use: Results of Logistic Regression Models Examining Age as a Moderator of the Associations Between Gender and Tobacco Use (N=2226).

| | **Smoking** | **Sada** | **Khainie** | **Liquid Tobacco** | **Betel Nut** |
|---|---|---|---|---|---|
| **Gender (Reference: Male)** | | | **AOR (95% CI)** | | |
| Female | 1.70 (1.08, 2.67) | 9.03 (6.44 × $10^{-9}$, 1.27 × $10^{-8}$) | 7.84 (3.99 × $10^{-8}$, 1.54 × $10^{-7}$) | 4.26 (2.02 × $10^{-7}$, 8.98 × $10^{-7}$) | 4.66 (0.25, 0.88) |
| Age categories (Reference: 18–24) | | | | | |
| 25–34 | 2.65 (1.36, 5.13) | 7.19 (3.80 × $10^{7}$, 1.36 × $10^{8}$) | 1.17 (1.17 × $10^{-4}$, 2.93 × $10^{-4}$) | 2.09 (2.09 × $10^{4}$, 6.58 × $10^{4}$) | 1.27 (1.27, 7.06) |
| 35–44 | 2.30 (1.26, 4.18) | 2.50 (1.21 × $10^{7}$, 5.13 × $10^{7}$) | 2.44 (2.44 × $10^{-5}$, 2.78 × $10^{-5}$) | 3.98 (3.98 × $10^{4}$, 5.75 × $10^{4}$) | 0.59 (0.59, 3.22) |
| 45–54 | 2.46 (1.33, 4.56) | 5.74 (3.14 × $10^{7}$, 1.05 × $10^{8}$) | 4.81 (4.81 × $10^{-5}$, 4.17 × $10^{-5}$) | 1.15 (1.15 × $10^{5}$, 1.04 × $10^{6}$) | 0.33 (0.33, 2.25) |
| 55+ | 1.20 (0.69, 2.09) | 2.23 (1.23 × $10^{7}$, 4.13 × $10^{7}$) | 4.63 (4.63 × $10^{-5}$, 3.01 × $10^{-6}$) | 4.93 (4.93 × $10^{5}$, 2.58 × $10^{6}$) | 0.21 (0.21, 1.23) |
| Gender * Age categories | | | | | |
| Female * 25–34 | 2.61 (0.29, 23.25) | 2.93 (1.49 × $10^{-8}$, 5.77 × $10^{-8}$) | 8.23 (1.21 × $10^{4}$, 3.06 × $10^{4}$) | 1.17 (2.65 × $10^{-6}$, 1.01 × $10^{-4}$) | 2.18 (0.19, 1.59) |
| Female * 35–44 | 1.20 (0.11, 12.67) | 6.22 (4.16 × $10^{-8}$, 1.89 × $10^{-7}$) | 2.52 (1.40 × $10^{-6}$, 1.78 × $10^{-5}$) | 1.51 (5.88 × $10^{-6}$, 9.97 × $10^{-5}$) | 1.28 (0.27, 2.32) |
| Female * 45–54 | 0.63 (0.63, 49.29) | 6.21 (3.31 × $10^{-8}$, 1.25 × $10^{-7}$) | 2.25 (8.21 × $10^{-7}$, 8.78 × $10^{-6}$) | 2.51 (7.72 × $10^{-6}$, 8.22 × $10^{-5}$) | 0.80 (0.68, 6.93) |
| Female * 55+ | 1.32 (1.32, 91.26) | 9.68 (3.21 × $10^{-8}$, 1.20 × $10^{-7}$) | 8.27 (2.75 × $10^{-7}$, 2.72 × $10^{-6}$) | 9.68 (9.68 × $10^{-6}$, 5.41 × $10^{-5}$) | 5.15 (0.81, 7.01) |

Note. Adjusted Odds Ratio = AOR. Confidence Interval = CI.
